# Magnetic Resonance Spectroscopy in Schizophrenia: Evidence for Glutamatergic Dysfunction and Impaired Energy Metabolism

**DOI:** 10.1007/s11064-018-2521-z

**Published:** 2018-04-03

**Authors:** João M. N. Duarte, Lijing Xin

**Affiliations:** 1 Department of Experimental Medical Science, Faculty of Medicine, Lund University, BMC C11, Sölvegatan 19, 221 84 Lund, Sweden; 2 Wallenberg Center for Molecular Medicine, Lund University, Lund, Sweden; 3 Animal Imaging and Technology Core (AIT), Center for Biomedical Imaging (CIBM), Ecole Polytechnique Fédérale de Lausanne, Lausanne, Switzerland

**Keywords:** Glutamate, Glutamine, Schizophrenia, Metabolism, Magnetic resonance

## Abstract

In the past couple of decades, major efforts were made to increase reliability of metabolic assessments by magnetic resonance methods. Magnetic resonance spectroscopy (MRS) has been valuable for providing in vivo evidence and investigating biomarkers in neuropsychiatric disorders, namely schizophrenia. Alterations of glutamate and glutamine levels in brains of schizophrenia patients relative to healthy subjects are generally interpreted as markers of glutamatergic dysfunction. However, only a small fraction of MRS-detectable glutamate is involved in neurotransmission. Here we review and discuss brain metabolic processes that involve glutamate and that are likely to be implicated in neuropsychiatric disorders.

## Introduction

Schizophrenia is a heterogeneous neurodevelopmental disorder affecting multiple domains of brain function, resulting in positive symptoms (e.g. delusions, hallucinations), negative symptoms (e.g. apathy, emotional blunting, low motivation), and cognitive symptoms (e.g. deficits in memory, attention and problem solving), which typically develop in late adolescence or early adulthood. While many sites in the human genome show association with the risk of developing schizophrenia, this disorder results from a complex interaction of the genetic predisposition with environmental and epigenetic factors [1].

A long-standing hypothesis of schizophrenia considers that dopamine depletion in the mesocortical neurons projecting from the ventral tegmentum to the prefrontal cortex results in feeble stimulation of neuronal dopamine D1 receptors, and contributes to negative symptoms and cognitive impairments. On the other hand, excessive activation of the mesolimbic pathway (projecting to the nucleus accumbens) results in overstimulation of D2 receptors, contributing to positive symptoms of schizophrenia. Antipsychotic treatments (e.g. haloperidol, clozapine) mainly target dopamine D2 receptors and effectively alleviate positive symptoms. However, negative symptoms and cognitive impairments are virtually irresponsive to typical antipsychotics because they involve other neurotransmission systems, namely those operated by glutamate and γ-aminobutyric acid (GABA), and are furthermore caused by a neurodegenerative process (discussed in [2, 3]). Therefore, a hypothesis suggesting that *N*-methyl-d-aspartic acid (NMDA) receptor hypofunction and inadequate GABAergic transmission play a major role in the disease has emerged [2, 4]. Normally, inhibitory interneurons monitor levels of excitatory neurotransmission via NMDA receptor signalling, and activated interneurons release GABA that acts on pyramidal neurons (glutamatergic) to achieve adequate excitatory–inhibitory balance. Due to NMDA receptor hypofunction, there is reduced NMDA receptor signalling in schizophrenia, which leads to a disruption of excitation monitoring, and thus GABAergic neurons respond as if excitatory transmission is insufficient. This poor negative feedback from GABAergic interneurons to pyramidal neurons results in increased glutamatergic neurotransmission that, in turn, leads to excitotoxicity. This hypothesis was supported by experiments on healthy humans that develop schizophrenia-like symptoms upon administration of ketamine at sub-anaesthetic doses [5]. Nowadays, there are several lines of evidence that support the NMDA receptor hypothesis: (1) NMDA receptor blockade causes symptoms of schizophrenia; (2) neuropathological studies identified a schizophrenia-associated reduction of presynaptic markers for GABAergic interneurons in the hippocampus and the intermediate layers of the prefrontal and cingulate cortex, namely reduced expression and/or density of GABA transporters (GAT), glutamic acid decarboxylase GAD67 (but not GAD65), and the Ca^2+^-binding protein parvalbumine; (3) several genes implicated in schizophrenia are involved in regulating NMDA receptors [2, 6, 7].

Glutamatergic neurotransmission acts on dopaminergic neurons. Thus, excessive firing of disinhibited pyramidal neurons can eventually drive the increase in dopamine release that is responsible for the positive symptoms of schizophrenia. Indeed, NMDA receptor inhibition in the prefrontal cortex increases extracellular glutamate that acts on AMPA and kainate receptors, which in turn stimulate dopamine release in the prefrontal cortex and striatum ([8] and references therein). Also in humans, sub-anaesthetic doses of ketamine stimulate striatal release of dopamine [9]. Although NMDA receptor hypofunction in schizophrenia may be considered as a primary event leading to exacerbated dopaminergic transmission [2–4, 6, 7], it remains unknown whether it is cause or consequence of GABAergic deficits.

**Fig. 1:**
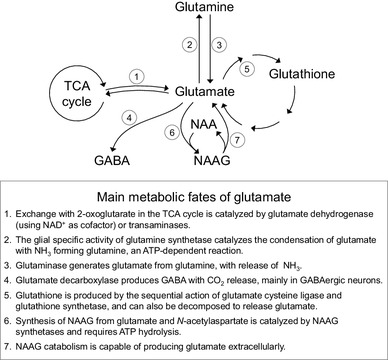
Main pathways of glutamate metabolism in the brain. Glutamate, glutamine, GABA, *N*-acetylaspartate, NAAG, and glutathione are detectable by ^1^H MRS

Glutamatergic and GABAergic systems may be investigated in vivo using magnetic resonance (MR) methods. In particular, the concentrations of these neurotransmitters, as well as products of their metabolism (Fig. 1), are detectable by ^1^H magnetic resonance spectroscopy (MRS) among several other metabolites that compose the so-called neurochemical profile [10]. Notably, MRS has served to investigate metabolic impairments in a variety of neurodegenerative disorders [11], and has also been employed to investigate biomarkers in psychiatric disorders, with strong incidence on schizophrenia [12]. However, one notes a particular inconsistency in the reported neurochemical alterations from MRS studies on schizophrenia patients, which may be attributed to the intrinsic heterogeneity of the disorder.

In this work, we reviewed recent literature reporting MRS studies in the realm of schizophrenia. Rather than an extensive literature review, we focused on recent studies on schizophrenia patients and animal models that are relevant for supporting the glutamatergic dysfunction hypothesis. We further discuss the involvement of glutamatergic players on brain metabolism pathways.

### MRS in Schizophrenia

**Fig. 2:**
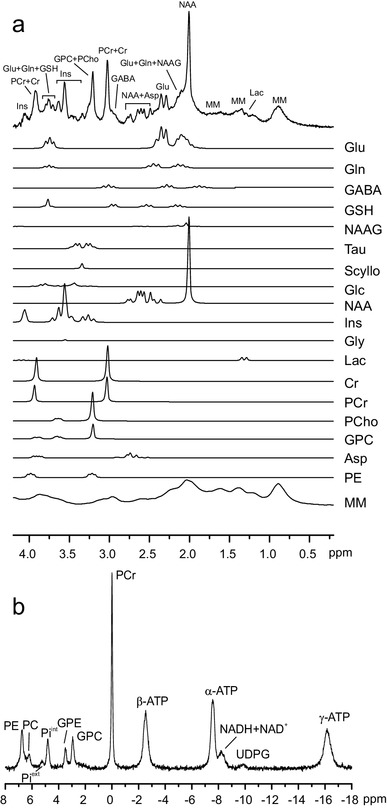
**a**^1^H MR spectrum of the human medial prefrontal cortex acquired with the SPECIAL sequence at 3 T (TE/TR = 6/4000 ms, number of averages = 148) and the fits of individual metabolites including aspartate (Asp), phosphocreatine (PCr), creatine (Cr), γ-aminobutyric acid (GABA), glutamine (Gln), glutamate (Glu), phosphorylcholine (PCho), glycerophosphorylcholine (GPC), glutathione (GSH), glucose (Glc), lactate (Lac), glycine (Gly), *myo*-inositol (Ins), *N*-acetylaspartylglutamate (NAA), *N*-acetylaspartylglutamate (NAAG), phosphorylethanolamine (PE), *scyllo*-inositol (Scyllo), taurine (Tau) and macromolecules (MM). **b**^31^P MR spectrum of the human occipital lobe at 7 T (a pulse-acquire sequence, spectral bandwidth = 6000 Hz, TR = 3 s, 320 averages, with baseline correction, no apodization). *PCr* phosphocreatine, *ATP* adenosine triphosphate, *Pi*^*int*^ intracellular inorganic phosphate, *Pi*^*ext*^ extracellular inorganic phosphate, *PE* phophothanolamine, *PC* phosphocoline, *GPC* glycerophosphocholine, *GPE* glycerophosphoethanolamine, *NADH* reduced form of nicotinamide adenine dinucleotide, *NAD*^+^ oxidized form of nicotinamide adenine dinucleotide, *UPDG* uridine diphosphoglucose

MRS allows the non-invasive measurement of stationary and dynamic information in humans and animals, i.e. concentrations of neurochemical compounds and metabolic fluxes, using MR-active nuclei such as ^1^H, ^31^P and ^13^C. ^1^H is the most sensitive nucleus for applications in living tissues and localized ^1^H MRS provides the measurement of about 20 metabolites in the brain including those related to neurotransmission, namely glutamate (Glu), glutamine (Gln), GABA, aspartate (Asp), *N*-acetylaspartylglutamate (NAAG), glycine (Gly) and serine; energy metabolism, which includes phosphocreatine (PCr), creatine (Cr), glucose (Glc), lactate (Lac) and alanine (Ala); phospholipid precursors involved in membrane metabolism, particularly phosphorylcholine (PCho), glycerophosphorylcholine (GPC), phosphorylethanolamine (PE); the antioxidants glutathione (GSH) and ascorbate (Asc); major osmolytes namely *myo*-inositol (Ins; *scyllo*-inositol is often also quantified) and taurine (Tau); finally the putative neuronal marker *N*-acetylaspartate (NAA) that is a precursor of NAAG (Fig. 2a). ^31^P MRS allows the measurement of energy related metabolites, namely ATP, phosphocreatine and inorganic phosphate (P_i_); membrane related phospholipid precursors including phosphomonoesters (PME): phosphocholine + phosphoethanolamine, reflecting membrane phospholipid synthesis, and phosphodiester (PDE): glycerophosphorylcholine + glycerophosphorylethanolamine, a marker of membrane degradation (Fig. 2b). Moreover, from the ^31^P spectrum one can estimate the intra-/extra-cellular pH and free Mg^2+^ concentration. ^13^C MRS is a method with low sensitivity due to its low natural abundance and gyromagnetic ratio. However, in combination with the infusion of specific ^13^C labelled substrates, it offers a unique way of measuring energy metabolic rates and neurotransmission in vivo [13, 14]. Due to the technical complexity such as substrate infusions and radiofrequency power deposition, and the high cost of ^13^C labelled compounds, its application in humans is limited as compared to animals. So far no ^13^C MRS study in schizophrenia patients was reported.

#### *N*-Acetylaspartate

*N*-Acetylaspartate is a putative marker of neuronal integrity, and is the most abundant metabolite in the central nervous system of adult mammals observed in ^1^H MR spectra [10, 15]. Therefore, its measurement in vivo by ^1^H MRS is rather straightforward and observed results are more consistent in schizophrenia patients. Decrements of *N*-acetylaspartate levels were found across different brain regions of schizophrenia patients relative to healthy controls, namely in frontal lobe, hippocampus and thalamus [16, 17]. With the disease progression, the degree of reductions in *N*-acetylaspartate tends to accentuate in patients with chronic schizophrenia (versus first episode patients; [17]). However, others have demonstrated increased *N*-acetylaspartate levels in hippocampus of chronic patients [18] and prefrontal cortex of high-risk adolescents [19]. Bustillo et al. reported recently that, with age, *N*-acetylaspartate increases in cortical grey matter and decreases in white matter of schizophrenia patients [20]. A reduction of brain *N*-acetylaspartate concentration with age and disease duration was found in a meta-analysis by Brugger et al. [21], and it is likely to reflect loss of neuronal metabolic integrity.

#### Glutamate and Glutamine

Alterations have also been abundantly reported for glutamine and glutamate, pointing towards schizophrenia-induced concentration changes in a time dependent manner: increased glutamine, glutamate and/or the ratio of glutamine-to-glutamate (Gln/Glu) have been found in early stages of the disease and unmedicated patients [22–27], whereas decreased levels of these amino acids have often been observed in chronic patients [26, 28–31]. A recent meta-analysis suggested a decline with age and disease duration in the levels of glutamate and glutamine [17].

Note that ^1^H MRS offers the measurement of neurochemical information at rest condition, while performing ^1^H MRS measurement during a functional task so called functional MRS (fMRS) allows the unique measurement of dynamic information of glutamatergic and energy metabolism upon neuronal activation. In fMRS studies, glutamate increase is a common manifestation of neuronal activation that has been interpreted as a response to stimulated energy metabolism [32, 33] because brain net amino acid synthesis requires increased anaplerotic activity, namely through pyruvate carboxylation [34]. These changes in glutamate levels are very subtle, and therefore such experiments require high sensitivity and stability of the MRS measurement. Recently, Taylor et al. conducted a fMRS study in schizophrenia patients at 7 T and, interestingly, they observed a delayed increase of glutamate levels in response to a cognitive task, comparing to healthy controls [35]. This suggests a neurodegeneration-like effect involving glutamatergic neurotransmission and oxidative metabolism.

#### GABA

Converging evidence suggests that abnormal function of GABAergic parvalbumin-positive interneurons leading to a loss of the balance between neuronal excitation and inhibition, and to deficits in neuronal synchronization, may contribute to cognitive deficits in schizophrenia. Accordingly, a reduction of mRNA encoding for the GABA synthesis enzyme GAD67 and aberrant gamma waves [36, 37] were observed in schizophrenia patients [38, 39]. The measurement of excitatory neurotransmitter glutamate in vivo by ^1^H MRS is straightforward due to its high cerebral concentration, on the contrary, the low level of GABA makes it challenging to be measured in vivo. Therefore, the number of studies of GABA by ^1^H MRS is very limited and most studies were conducted by spectral editing methods at 3 T [40–46], at 4 T [47], and recently also at 7 T [26, 48, 49]. A comprehensive meta-analysis shows that no significant schizophrenia-associated changes in GABA levels can be identified in medial prefrontal cortex, parietal/occipital cortex and striatum, which is not consistent with the post-mortem studies indicating a reduction in GABA synthesis [38, 39]. However, this may be attributed to the diverse methodologies used, the regional specificities or the compensation of other unaffected subtype of interneurons [42].

#### Glutathione

Glutathione is a major cellular redox regulator and antioxidant protecting cell from damages induced by reactive oxygen species. Its levels are decreased in cerebrospinal fluid and medial prefrontal cortex of chronic schizophrenia patients [50] and the lower glutathione levels are associated with more severe negative symptoms [51]. Moreover, subjects carrying polymorphisms in the gene coding for the catalytic subunit of the glutamate–cysteine ligase (*Gclc*) that are associated with high risk of developing schizophrenia [52, 53] display lower glutathione concentrations in the medial prefrontal cortex than low-risk genotype subjects [54]. Interestingly, low prefrontal glutamate levels are present in patients with low-risk genotypes [54], suggesting a predominant pathogenic role of glutamatergic system impairments in *Gclc* low-risk genotypes. However, unchanged cerebral glutathione levels were also reported between patients and controls [54, 55]. This may be linked to the different distributions of *Gclc* polymorphisms between groups and/or the measurement under resting conditions, where alterations may not be pronounced as under particular conditions, such as upon psychosocial stress exposure. However, stress-induced glutathione alterations remain to be directly demonstrated in patients or subjects at risk of developing schizophrenia. In addition, glutathione was also shown to associate with white matter integrity and resting-state functional connectivity along the cingulum bundle, and this association with functional connectivity seems to be disrupted in early psychosis patients [56].

#### NAD^+^/NADH

The equilibrium between the oxidized (NAD^+^) and reduced (NADH) forms of nicotinamide adenine dinucleotide play a key role in many biological processes such as energy metabolism, antioxidation, and calcium homeostasis, and their ratio reflects cellular redox state. Their measurement in vivo is challenging due to its low concentration and limited spectral separation. With the increase of sensitivity and spectral dispersion at high magnetic fields, and with the improvements made for spectral fitting, recent studies have reported the measurement of NAD^+^ and NADH in human brains in vivo by ^31^P MRS [57]. One application of this method revealed a significant reduction of redox state NAD^+^/NADH in both early psychosis and chronic patients, offering for the first time direct in vivo support of the presence of redox imbalance in schizophrenia patients, possibly linked to mitochondrial dysfunction and impaired energy metabolism [58].

#### Mitochondrial Dysfunction

Abnormalities in mitochondrial function and energy metabolism have also been proposed to occur in patients with schizophrenia [59]. Although as shown by ^31^P MRS studies no consistent changes of ATP, phosphocreatine and P_i_ can be identified from frontal lobe, temporal lobe and subcortical regions [60], interestingly the reaction rate of creatine kinase, the enzyme catalysing the exchange between phosphocreatine and ATP, was decreased in anterior cingulate cortex of patients using magnetization transfer experiment in ^31^P MRS [61]. Apart from methodological heterogeneities (discussed in [60]), the potential compensatory mechanism at rest likely plays a role such that dynamic change can be observed in the absence of changes in total resting pool size. In addition, accumulation of lactate was observed in medial prefrontal cortex [62] and cerebral spinal fluid [63] in schizophrenia patients, suggest a shift towards reliance on glycolysis for energy production, and may lead to lactic acidosis. Accordingly, low brain pH values in schizophrenia patients were observed in one ^31^P MRS study [61].

Taken together, the observations in patients by MRS studies indeed offer in vivo evidences supporting the presence of glutamatergic, redox and mitochondrial dysfunction in schizophrenia, despite many confounding factors involved such as MR methodologies, disease stage, region specificity, heterogeneity in patient cohorts and medication effects. It is important to note that compensatory mechanisms at resting state may mask changes in metabolite concentrations. In contrast, dynamic MRS measurements may be more informative, as is the case of fMRS studies [35] and magnetization transfer experiments [61] in patients.

Furthermore, one should note that only small fractions of the measured pools of glutamate and GABA are involved in neurotransmission. To directly address the aberrant glutamate and GABA neurotransmission, as well as energy metabolism pathways, future studies using ^13^C MRS are required [13] and are expected to provide novel insights in pathophysiology of schizophrenia.

### MRS on Animal Models: Glutamate/Glutamine Alterations

MRS can be applied in both clinical and pre-clinical settings, and thus represents a valuable method for translational research. The establishment of animal models for schizophrenia (and for the so complex psychiatric disorders in general) is challenging and does not fully recapitulate all behavioural phenotypes of the disease. Recent research on animal models has focused particularly on mice carrying modifications in genes associated to the risk of developing schizophrenia, as well as on the exposure to environmental insults. MRS has also been employed to identify metabolic modifications triggered by acute administration of NMDA receptor antagonists that mimic behavioural symptoms similar to those observed in schizophrenia (e.g. the open channel blockers phencyclidine, ketamine). In this section we assembled evidence from studies on animal models of schizophrenia and of psychological stress that triggers schizophrenia-like phenotypes, which might contribute to explain findings in the brain of schizophrenia patients.

Phencyclidine was reported to acutely induce a reduction of glutamate levels accompanied by glutamine accumulation (without changes in the total content of glutamate plus glutamine, called “Glx”) in the rat prefrontal cortex [64]. This MRS study at high magnetic field supports changes in Gln/Glu as a putative marker for the glutamatergic dysfunction in schizophrenia. Psychosocial stress is a major trigger of neuropsychiatric disorders [65–67]. In rodents, exposure to stress early in life causes anxiety and depressive-like behaviours, and might also contribute to the development of schizophrenia-like behaviours [68–70]. Interestingly, Napolitano et al. reported that social isolation, an often used psychosocial stress paradigm, results in an altered response to a NMDA receptor non-competitive antagonist (ketamine) challenge in mice, namely an exacerbated ketamine-induced glutamine increase and a reduction of GABA concentration in the prefrontal cortex [71]. In particular, exposing rodents to social isolation stress after weaning leads to mitochondrial dysfunction and increased oxidative stress [69, 72], impaired function of oligodendrocytes that results in poor myelination [73, 74], and degeneration of parvalbumine-positive neurons and neuroinflammation [75] within the prefrontal cortex. These features are likely to cause alterations of the neurochemical profile measured by MRS. In a recent study, we observed that social isolation after weaning causes an increase of Gln/Glu, as well as a reduction of *myo*-inositol in the mouse frontal cortex [76]. In addition, recent work suggests that a number of stress paradigms applied to mice early in life leads to reduced concentration of metabolites produced and stored in neurons, namely glutamate, GABA and *N*-acetylaspartate, almost exclusively in the prefrontal cortex ([77] and references therein). Also Vernon et al. reported altered metabolite concentrations in the prefrontal cortex of adult mice born from females exposed to immune activation during gestation, namely decreased levels of glutathione, taurine and *N*-acetylaspartate [78].

As discussed above, reduced levels of glutathione are a recurrent observation in the brains of schizophrenia patients, and genes involved in glutathione synthesis have been implicated in the disease [79]. Mice with a functional deletion in the modulatory subunit of the glutamate–cysteine ligase (*Gclm*) display impaired glutathione synthesis leading to reduced glutathione levels in multiple organs, including the brain [80, 81]. Compared to wild-type mice, *Gclm* −/− mice were reported to have delayed oligodendrocyte maturation (which depends on adequate intracellular redox balance) and myelination in the anterior cingulate cortex, and impaired white mater integrity [56, 82]. Furthermore, early-life insults inducing oxidative stress in *Gclm* −/− mice are detrimental to immature parvalbumin-immunoreactive interneurons and have consequences for anterior cingulate cortex functioning in adulthood [83]. Elevated Gln/Glu was also found in the frontal cortex of the *Gclm* −/− mouse relative to controls [82, 84], which is further accentuated by social isolation stress after weaning [76]. In line with these observations, glutamine synthetase was suggested to be impacted by oxidative stress [85], which occurs in neuropsychiatric disorders and upon NMDA receptor inhibition [78, 86]. Interestingly, a mouse model of schizophrenia induced by perinatal treatment with ketamine was reported to display a persistent reduction of glutathione and increased oxidative stress in the medial prefrontal cortex [87], which was accompanied by impaired mitochondrial function, reduced parvalbumin expression, and disruption of the excitation–inhibition balance (reduced inhibition onto layer 2/3 pyramidal cells, and increased excitatory drive onto parvalbumin-positive interneurons). These alterations were ameliorated by *N*-acetylcysteine treatment [87], a compound that might stimulate glutathione synthesis and also acts as antioxidant [84, 87].

### Cerebral Energy Metabolism and Glutamatergic Function

#### Glutamatergic Neurotransmission and Signalling Through NMDA Receptors

Given its relatively large concentration in the brain (~ 10 mM in cortex), glutamate is of easy quantification by MRS [10], and has been proposed as a biomarker for neurodegeneration in several pathologies [11]. However, studies at low magnetic field are unable to distinguish between glutamate and glutamine signals and have reported the sum of their concentrations, called “Glx”.

Glutamate is the main excitatory neurotransmitter in the brain and plays a major role in psychopathology. Synaptic vesicles in nerve terminals of glutamatergic neurons are loaded with glutamate. Following an action potential, glutamate is released via exocytosis. Once in the synaptic cleft, it can activate ionotropic (ion channels) and metabotropic (GTP-binding protein coupled) receptors. The ligand-gated ion channels are further divided into three families: α-amino-3-hydroxy-5-methyl-isoxazole-4-propionate (AMPA), kainate and *N*-methyl-d-aspartate (NMDA). In the mammalian central nervous system, AMPA and kainate receptors mainly mediate rapid depolarizing responses, the excitatory post-synaptic currents (EPSCs) [88]. The NMDA receptor participates mainly in synaptic plasticity and synapse formation, although it can also contribute to EPSPs and dendritic spikes [89]. The family of metabotropic receptors comprises several members within three subtypes, all involved in modulating synaptic signalling by glutamate and other transmitters [90].

As discussed above, NMDA receptors of the postsynaptic spine are of particular importance in the pathophysiology of schizophrenia. The NMDA receptor is a tetramer containing two subunits: NR1, which is required for channel function, and NR2A–D or NR3A,B, which determine biophysical and pharmacologic characteristics of the receptor (e.g. reviewed by [3]). NMDA receptors require that two different ligands bind to open the ion channel: glutamate binds to a site on the NR2/3 subunit (same site of NMDA and aspartate binding), and glycine, d-serine, kynurenic acid or NAAG binds to the modulatory binding site on the NR1 subunit. The channel also comprises a binding site for dissociative anaesthetics, including phencyclidine, dizocilpine (MK-801) and ketamine. At resting membrane potential, the NMDA receptor channel is blocked by Mg^2+^ that is released upon depolarization, allowing cation transport across the pore.

Glycine, Serine and NAAG are detectable in the brain by MRS, but their quantification may be hampered by the overlap with peaks from metabolites with much larger concentration, especially when operating at low magnetic fields [10] where spectral resolution is poor. However, specific editing techniques have been employed to resolve signals of these metabolites in ^1^H MRS [91]. NAAG is of particular interest for glutamatergic neurotransmission. In addition to acting as an antagonist of the glycine site of the NMDA receptor [92], NAAG is synthetized from glutamate (Fig. 1). It is a peptide widely spread in the nervous system and particularly concentrated in synaptic vesicles of presynaptic terminals, including those of pyramidal neurons in the cortex and limbic system, being co-released with glutamate [93]. NAAG is synthetized from ATP-dependent condensation of *N*-acetylaspartate and glutamate in neurons (Fig. 1) catalysed by NAAG synthetase activities [94, 95]. Although its role is not fully understood, NAAG does specifically interact with the NMDA receptor (but not with AMPA or kainate receptors) through which it modulates synaptic plasticity [93]. Therefore, the balance between synaptic levels of glutamate and NAAG, as well as other ligands of the glycine site, is important to modulate cation translocation through the receptor’s pore.

Importantly, NAAG also acts on the metabotropic type II glutamate receptor mGluR3 [96, 97] inhibiting the release of neurotransmitters, such as glutamate, GABA and glycine [93, 96–99]. Interestingly, it was reported that inhibitors of glutamate carboxypeptidase II (enzyme that inactivates *N*-acetylaspartylglutamate) are useful in rescuing memory impairments in rodent models of neurogenerative disorders [100], as well as preventing increases in glutamate release induced by the NMDA receptor non-competitive antagonist phencyclidine, through NAAG actions on mGluR3 [99]. Such evidence suggests that increasing levels of NAAG might help re-establishing the inhibitory–excitatory balance in schizophrenia.

Reduced cortical levels of NAAG have been reported in schizophrenia, as well as in bipolar disorder [101]. A deficit in NAAG levels would therefore (1) increase the activity of NMDA receptors, leading to over-activation of GABAergic interneurons and consequently disinhibit pyramidal neurons, (2) and reduce the agonist action on mGluR3 receptors located pre-synaptically in glutamatergic neurons, thus removing the negative feedback. Both actions result in increased glutamate release.

Although brain NAAG occurs in concentrations detectable by MRS at high magnetic field, its main resonances overlap with those of *N*-acetylaspartate and are thus of difficult analysis [10]. Therefore, the reduction of *N*-acetylaspartate levels reported in brains of schizophrenia patients (studies generally conducted at low magnetic field; see above) likely includes reduced NAAG.

#### Astrocytes are Capable of Regulating NMDA Receptor Activity

Glutamatergic signalling is terminated by an uptake mechanism that uses the driving force of Na^+^, K^+^ and H^+^ gradients to transport glutamate against its concentration gradient, keeping extracellular glutamate concentration below the level that activates its receptors (~ 1 µM; [102, 103]). This transport mechanism not only ensures adequate synaptic signalling but also prevents glutamate-mediated excitotoxicity (excessive glutamatergic activity) that triggers brain injury. Astrocytes are crucial for glutamatergic neurotransmission, particularly by clearing glutamate of neuronal origin in their processes abutting synapses, by modulating neuronal activity, and by controlling nutrient delivery from circulation ([34] and references therein).

**Fig. 3:**
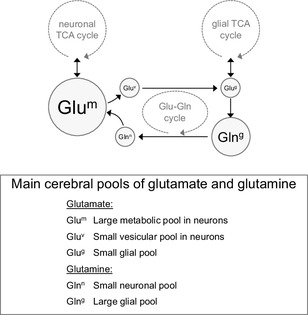
Relative sizes of the main cerebral pools of glutamate and glutamine linked to the TCA cycles of neurons and glial cells

Once glutamate is taken up by astrocytes, it can be either oxidised or converted to glutamine in an energy-dependent manner via the glial-specific enzyme, glutamine synthetase [104]. Thus, the pool of astrocytic glutamate is considered much smaller than that of neurons, while the opposite is proposed for glutamine compartmentation (Fig. 3; discussed in [105]). Glutamine, which is electrophysiologically inactive, is then shuttled to neurons, and the glutamate–glutamine cycle is finally completed by action of neuronal glutaminase and packing of the produced glutamate into vesicles. Therefore, it is generally considered that neurons include a large metabolic pool of glutamate separated from the pool that comprises vesicular glutamate. This is however an oversimplified view of metabolic compartmentation. It is important to note that both glutamate and glutamine can be diverted to a number of metabolic processes, such as the formation of GABA or glutathione, protein synthesis, or catabolism in the tricarboxylic acid (TCA) cycle (Fig. 1), which can result in multiple kinetically different glutamate pools. Also contributing to multiple pools is the intracellular tortuosity of diffusion paths that may render metabolic pools not fully interchangeable.

The involvement of astrocytes in regulating NMDA activity is of importance for schizophrenia. First of all, as mentioned above, astrocytic processes that engulf glutamatergic synapses are equipped with high density of the transporters that clear synaptic glutamate, and readily respond to glutamate release by engaging paracrine signalling and stimulating its metabolism (reviewed by [34]). Moreover, as expertly reviewed by others [2, 3, 93], astrocytes regulate the synaptic availability of NMDA receptor modulators: (1) kynurenic acid, an antagonist at the glycine binding site of the NMDA receptor, is produced from tryptophan and released by astrocytes; (2) astrocytes possess the glycine transporter type 1 that regulates synaptic levels of glycine; (3) both serine racemase that synthesizes d-serine, and d-amino acid oxidase that catabolizes d-serine are localized in astrocytes; (4) astrocytic end-feet contain glutamate carboxypeptidase II that catabolises NAAG to *N*-acetylaspartate and glutamate. Malfunctioning of any of these pathways in astrocytes is likely causing schizophrenia-like phenotypes. Nevertheless, the major role of astrocytes in regulating glutamatergic activity remains the clearance of synaptic glutamate and maintenance of the glutamate–glutamine cycle, which are energetically costly [34, 102].

Many studies found reduced levels of glutamate transporters in the brain of schizophrenia patients, which could contribute to glutamate accumulation after release into the synapse (e.g. [106, 107]; reviewed in [108]). A recent study analysed cell-specific expression of transporters, and reported that the density of glutamate transporters is reduced in astrocytes but not in neurons [109]. This suggests that the astrocytic support to glutamatergic neurotransmission is impaired. In addition, differential mRNA expression of splice variants of the excitatory amino acid transporters EAAT1 and EAAT2 was observed in pyramidal neurons of the anterior cingulate cortex in the schizophrenia post-mortem brain [110]. Also polymorphisms of EAAT1 and EAAT2 were proposed to be associated with cognitive performance in schizophrenia [111]. These findings provide an additional dimension to the regulation of glutamate transport that is relevant for schizophrenia but remains to be understood.

Another point of glutamatergic regulation by astrocytes is the glutamate dehydrogenase, a mitochondrial enzyme that catabolises glutamate to 2-oxoglutarate for mitochondrial further oxidation (see [34] and references therein). Reduced glutamate dehydrogenase activity and concomitant increased hippocampal glutamate levels were found in patients with mesial temporal lobe epilepsy, with SCZ-like psychotic symptoms and cognitive deficits [112]. However, inconsistent findings on glutamate dehydrogenase have reported in post-mortem analyses of brains from schizophrenia patients [113–115]. Interestingly, a brain-specific glutamate dehydrogenase knock-out mouse was reported to show schizophrenia-like behaviours, reduced hippocampal volume, disrupted hippocampal excitatory–inhibitory balance [115].

Both glutamate uptake by astrocytes and its mitochondrial catabolism initiated by glutamate dehydrogenase are important nodes of glutamatergic regulation within astrocytes, and alterations of these processes might contribute to metabolic deregulation in schizophrenia.

#### Energy Metabolism and the Glutamate–Glutamine Cycle

Brain function is demanding in terms of energetic requirements, as all transport of ions across cell membranes is dependent on ATP, the main cellular energy carrier. When ion translocation does not require direct ATP hydrolysis, this takes place upon subsequent reestablishment of the ionic gradients. Thus, glutamatergic dysfunction in schizophrenia is certainly associated to metabolic impairments.

Most of the cell’s ATP is produced by oxidative phosphorylation using reducing equivalents generated by the TCA cycle in the mitochondria. Therefore, both neurons and astrocytes stimulate their TCA cycle rates in response to increased activity, namely glutamatergic neurotransmission [105].

As discussed elsewhere [10], the pool of glutamate involved in synaptic transmission is much smaller than that associated to energy metabolism (Fig. 3). Thus, it is likely that alterations of energy metabolism in schizophrenia represent a major contribution to changes in the levels of cerebral glutamate and glutamine (as well as *N*-acetylaspartate that is produced in mitochondria; [15]) measured by MRS in patients relative to healthy subjects. Accordingly, in a study of the aging human brain, Boumezbeur and co-authors employed ^1^H and ^13^C MRS, and identified a correlation between levels of glutamate and *N*-acetylaspartate (taken as marker of neuronal integrity) and the TCA cycle rate in neurons, as well as between the levels of *myo*-inositol (a putative glial marker, discussed in [10]) and the TCA cycle rate in glial cells [116].

There is indeed abundant evidence pointing towards metabolic dysfunction in schizophrenia. Many studies found altered expression of genes related to brain energy metabolism in schizophrenia patients [117, 118]. In an interesting study that combined transcriptomics, proteomics and metabolomics on the prefrontal cortex of schizophrenia patients and controls (post-mortem), nearly half the altered proteins were associated with mitochondrial function, metabolism and oxidative stress, and were indeed associated with alterations at transcriptional and metabolic levels [119]. Regarding oxidative metabolism, several studies reported that schizophrenia alters the expression and activity of enzymes of the TCA cycle, as well as components of the mitochondrial electron transport chain and oxidative phosphorylation [120]. Compared to controls, reduced number and size of mitochondria have also been reported in neurons and oligodendrocytes of brains of schizophrenia patients (e.g. [121, 122]). In addition, impaired cerebral metabolic rate of glucose in schizophrenia patients was observed in positron emission tomography studies, namely in frontal areas [123, 124]. Studies of functional magnetic resonance imaging further suggest an impairment of cerebral blood flow and/or oxygen metabolism, consistent with a disruption of the neurovascular and/or neurometabolic coupling in frontal areas of patients with schizophrenia both at rest and during activation [125, 126].

Research in animal models confirmed such brain metabolic disturbances. Acute antagonism of NMDA receptors modulates mitochondrial oxidative metabolism, as was for example demonstrated in the rat (e.g. [127]). Mimicking persistent hypofunction of NMDA receptors, a sub-anaesthetic dose of ketamine administered to rats over a week resulted in impaired activities of mitochondrial respiratory chain complexes in the prefrontal cortex, striatum and hippocampus [128]. Rodents exposed to social isolation stress after weaning, which is recognised to contribute for developing anxiety, and depressive- and schizophrenia-like behaviours [68], also display mitochondrial dysfunction and increased oxidative stress in cortical areas [69, 72, 129].

However, such impairments in components of mitochondrial metabolism may not directly translate to alterations of metabolic fluxes in vivo due to potential compensatory mechanisms. As discussed above, glutamate release is tightly coupled to energy metabolism. According to the glutamatergic hypothesis of schizophrenia, reduction of NMDA receptor activity in inhibitory neurons results in disinhibition of pyramidal cells with concomitant stimulation of glutamate release, and an increase in oxidative metabolism is thus expected. Indeed, increased extracellular glutamate after systemic injections of NMDA receptor antagonists, such as the open channel blockers phencyclidine and ketamine, was observed using micro-dialysis in the prefrontal cortex [8, 130].

This released glutamate is likely taken by astrocytes and accumulated in the form of glutamine, which can be provided to neurons, and a glutamate–glutamine cycle rate increase is expected to result in stimulation of neuronal and astrocytic TCA cycle [105, 131]. Accordingly, a phencyclidine-induced glutamate reduction and glutamine increase were reported by Iltis et al. [64]. In a later study, Chowdhury et al. employed ^1^H-[^13^C] MRS to measure rates of global energy metabolism and the glutamate–glutamine cycle in the frontal cortex and hippocampus of rats acutely treated with ketamine. Indeed, a sub-anaesthetic, but not anaesthetic, dose of ketamine significantly increased the fraction of ^13^C labelling in carbons of glutamate, GABA and glutamine upon infusion of either [1-^13^C]glucose or the glial specific substrate [2-^13^C]acetate [132]. Altogether, these observations suggest that NMDA receptor antagonists (administered at a sub-anaesthetic dose) result in stimulation of the glutamate–glutamine cycle rate as well as of oxidative metabolism in both neurons and astrocytes within the prefrontal cortex.

It should be noted, however, that not all cortical areas are likely to present the same metabolic alterations in schizophrenia, as is evidenced for example in positron emission tomography studies [123, 124, 133]. In these lines, a recent study in a methylazoxymethanol acetate rat model of schizophrenia showed that resting oxidative metabolism, as measured by ^1^H-[^13^C] MRS with administration of [1,6-^13^C]glucose, was reduced in the orbitofrontal cortex, increased in the visual cortex, and unaltered in the somatosensory cortex and dorsal hippocampus, when compared to controls [134]. These observations were compatible with functional and structural connectivity differences and behavioural outcomes, which indicated hypofrontality and posterior hyperactivity [134].

Changes of energy metabolism require a direct match of glucose utilisation rates. Upon phencyclidine administration, Iltis and co-workers observed an immediate rise in cortical glucose levels that normalised within 30 min, while lactate content was substantially reduced and took nearly 1 h to reach baseline levels [64]. These observations are compatible with an initial reduction of glycolysis upon acute phencyclidine administration. Accordingly, glucose utilisation was found decreased within some cortical areas after 2 min of phencyclidine administration [135]. Notably, 1–3 h after administration of NMDA receptor antagonists, increased glucose utilisation was shown in the prefrontal cortex, as well as in other cortical areas (mainly layers 1–3) and in parts of the limbic system [135, 136], which is consistent with increased metabolism in neurons and astrocytes [132]. Also in humans, NMDA receptor blockade with sub-anaesthetic doses of ketamine enhances blood flow and glucose utilisation in the prefrontal cortex and anterior cingulate cortex [137–139].

Investigation of brain energy metabolism in schizophrenia patients has been attempted. In particular, as noted above, ^31^P MRS has been used to measure the content of phosphorus-containing compounds in the brain of schizophrenia patients, including ATP and phosphocreatine that are related to energy metabolism [60]. Although few ^31^P MRS studies showed reduced levels of these energy-related metabolites in cortical areas of patients relative to healthy subjects, a comprehensive review of the available literature led Yuksel and co-authors to observe that available data is highly variable and there are inconclusive in terms of metabolic alterations. First, ^31^P MRS studies typically have small sample sizes, which masks potential schizophrenia-induced alterations. Another major concern is the lack of control on the medication effects and illness progression. Finally, it is clear that methodological improvements are required for standardisation of the acquired data: studies should be conducted at high magnetic field strengths to increase sensitivity and spectral resolution; and there should be a correction for the tissue composition (white matter, grey matter, cerebral spinal fluid) of the volume-of-interest used for signal detection because it is known to change with disease progression.

It is likely that brain homeostasis is maintained, thus resulting in unaltered baseline metabolite concentrations, whereas the response to a stress challenge of a complex, energetically demanding task may reveal specific metabolic impairments (discussed in [77]). In a recent study on bipolar disorder patients, ^31^P MRS was performed in the visual cortex during a visual stimulation task [140]. While there were no ATP or phosphocreatine abnormalities at baseline, there were distinct stimulation-induced metabolic changes in patients and healthy controls: visual stimulation reduced the levels of phosphocreatine but not ATP in controls, and reduced the levels of ATP but not phosphocreatine in patients. These results suggest that the visual cortex of bipolar disorder patients is limited in recruiting phosphocreatine as energetic buffer through creatine kinase.

The rate of creatine kinase can be directly measured in vivo using magnetisation transfer technics combined with ^31^P MRS. In the frontal cortex of schizophrenia patients, Du et al. [61] reported a significant reduction of the creatine kinase reaction rate, while phosphocreatine and ATP levels were normal at baseline.

Interestingly, reduced glucose tolerance and impaired insulin sensitivity have been reported in schizophrenia patients and their siblings, relative to healthy subjects [141–143], which suggests that metabolic dysfunction in schizophrenia is not exclusive to the brain. It should be noted as well that impaired metabolism and mitochondrial dysfunction were also suggested to occur in other psychiatric disorders such as bipolar disorder [144, 145], major depressive disorder [146] or autism [147].

Metabolic impairments linked to mitochondrial dysfunction are accompanied by oxidative stress, which is an important component of schizophrenia. As reviewed elsewhere [79, 86], impairments in redox homeostasis and susceptibility of increased oxidative stress are linked to glutamatergic dysfunction due to (1) hypoactive NMDA receptors, (2) to degeneration of fast-spiking parvalbumin-positive GABAergic interneurons that are essential for fast local neuronal synchronization, (3) to dysfunctional oligodendrocytes resulting in poor myelination and thus impairing axonal integrity and signal conduction across brain areas, and (4) to neuroinflammation.

#### Glutamate as a Marker for Neuronal Density

Glutamate is the most concentrated amino acid in the brain. As discussed above, glutamine synthetase resides exclusively in glial cells, and neurons readily convert glutamine into glutamate (Fig. 3). Thus, while most glutamate resides in neurons of the mammalian brain, glutamine is thought to be mainly localized to astrocytes (for revision see [34]). Therefore, a reduction in the size or amount of space occupied by neurons, relative to the volume occupied by other brain cells, might result in a reduction of glutamate concentration relative to that of glutamine.

In the *Gclm* −/− mouse model of schizophrenia, we found increased Gln/Glu in the frontal cortex (relative to both +/+ and +/− mice), without a reduction of glutamate but an increase in glutamine levels [76, 82, 84]. Moreover, social isolation in mice was also found to cause an increase in the ratio of glutamine-to-glutamate (Gln/Glu) in the frontal cortex and a trend towards a decrease in glutamate concentration [76]. Since glutamate is primarily located in neurons, neuronal loss or reduced neuronal processes are likely to result in decreased tissue glutamate content [10]. Supporting this notion, chronic stress was reported to result in a marked reduction of the dendritic arborisation in the medial prefrontal cortex [148–150]. Chronic social isolation stress was also shown to reduce levels of glutamate receptors in cortical areas and the hippocampal formation [151–153], suggesting impairments in glutamatergic neurons.

Increased Gln/Glu was also reported in the cerebrospinal fluid of schizophrenia patients, relative to healthy subjects [23]. Bustillo et al. reported higher anterior cingulate Gln/Glu in minimally treated patients than in control subjects [24]. Interestingly, glutamate was reported to be higher in young schizophrenia patients, but to decrease with age, relative to healthy subjects [17, 26].

Neurons are also the primary reservoir of *N*-acetylaspartate. The concentration of *N*-acetylaspartate is relatively high in neurons and, since it is synthetized in neuronal mitochondria and endoplasmic reticulum [15], it is sensitive to mitochondrial dysfunction and deleterious effects of oxidative stress, being considered a marker of neuronal health (reviewed in [10]). Interestingly, since *N*-acetylaspartate hydrolysis by aspartylacylase in oligodendrocytes and astrocytes produces oxaloacetate and acetate that are substrates for the TCA cycle, it can serve either for fuelling activity of these glial cells or for de novo glutamate synthesis [154]. Impairments of aspartylacylase activity are thus likely to result in increased *N*-acetylaspartate levels without improved mitochondrial function in neurons (e.g. [155]).

Correlation between concentrations of *N*-acetylaspartate and glutamate is observable in the brains of both rodents [156] and humans [157]. This correlation between the neuronal markers *N*-acetylaspartate and glutamate has been found disrupted in frontal areas of patients with neuropsychiatric disorders, including schizophrenia (e.g. [158, 159]). Importantly, in schizophrenia, glutamate loss was found to correlate with functional deterioration and with grey matter loss [160]. This is consistent with glutamate being a marker for neurodegeneration.

## Conclusion

Although changes in glutamate levels in schizophrenia have been linked to impaired glutamatergic neurotransmission and hypofunction of NMDA receptors, it is likely that glutamate alterations also result from metabolic impairments, particularly at mitochondrial level. Moreover, given the primary location of glutamate in neurons and glutamine in astrocytes, one must consider that deterioration of neuronal processes, reduction of dendritic arborisations, etc. might result in increased Gln/Glu.

## Abbreviations

AMPA: α-Amino-3-hydroxy-5-methyl-isoxazole-4-propionate
ATP: Adenosine triphosphate
EPSCs: Excitatory post-synaptic currents
GABA: γ-Aminobutyric acid
Glx: Glutamate plus glutamine
mGluR2/3: Metabotropic glutamate receptors 2 or 3
MR: Magnetic resonance
MRS: Magnetic resonance spectroscopy
NAAG: *N*-Acetylaspartylglutamate
NMDA: *N*-Methyl-d-aspartate
TCA: Tricarboxylic acid
